# Women’s health and well-being in five birth cohorts from low- and middle-income countries: Domains and their associations with early-life conditions

**DOI:** 10.7189/jogh.14.04137

**Published:** 2024-08-16

**Authors:** Fernando Pires Hartwig, Anushka Ataullahjan, Linda Adair, Helen Gonçalves, Bernardo Horta, Nanette Lee, Reynaldo Martorell, Ana Maria B Menezes, Janaina Vieira dos Santos Motta, Shane Norris, Manuel Ramirez-Zea, Linda Richter, Zulfiqar Bhutta, Aryeh D Stein, Cesar Victora, COHORTS consortium

**Affiliations:** 1 Postgraduate Program in Epidemiology, Federal University of Pelotas, Pelotas, Brazil; 2 Centre for Global Child Health, Hospital for Sick Children, Toronto, Ontario, Canada; 3 Department of Nutrition, Gillings School of Global Public Health, University of North Carolina, Chapel Hill, North Carolina, USA; 4 USC Office of Population Studies Foundation, University of San Carlos, Cebu, Philippines; 5 Hubert Department of Global Health, Rollins School of Public Health, Emory University, Atlanta, Georgia, USA; 6 SAMRC Pathways for Health Research Unit, School of Public Health, Faculty of Health Sciences, University of the Witwatersrand, Johannesburg, South Africa; 7 INCAP Research Center for the Prevention of Chronic Diseases, Institute of Nutrition of Central America and Panama, Guatemala City, Guatemala; 8 Department of Science and Innovation, National Research Foundation Centre of Excellence in Human Development, School of Public Health, Faculty of Health Sciences, University of the Witwatersrand, Johannesburg, South Africa; 9 Institute for Global Health and Development, The Aga Khan University, Karachi, Pakistan; 10 International Center for Equity in Health, Federal University of Pelotas, Pelotas, Brazil

## Abstract

**Background:**

Women’s health and well-being (WHW) have been receiving growing attention, but limited progress has been made on how to measure its different domains in low- and middle-income countries (LMICs). We used data from five long-term birth cohorts in Brazil, Guatemala, the Philippines and South Africa to explore different domains of adult WHW, and how these domains relate to early life exposures.

**Methods:**

Based upon an a priori conceptualisation of eight postulated WHW outcomes available in the data, we grouped them as follows: human capital (intelligence quotient, schooling, height, and teenage childbearing), metabolic health (body mass index and metabolic syndrome score), and psychological (happiness and Self-Reported Questionnaire (SRQ) scores). Correlation analyses confirmed the variables theoretically belonging to the same dimension of WHW were statistically related. We then applied principal component analysis to each group of variables separately and used the first principal component as a summary quantitative measure of the corresponding WHW dimension. Finally, we assessed the association of each domain with a range of early-life factors: wealth, maternal education, maternal height, water, and sanitation, birthweight, length at two years and development quotient in mid-childhood.

**Results:**

The three domains were largely uncorrelated. Early determinants were positively associated with human capital, while birth order was negatively associated. Fewer associations were found for the metabolic or psychological components. Birthweight and weight at age two years were inversely associated with metabolic health. Maternal education was associated with better psychological health.

**Conclusions:**

Our findings indicate that WHW is multidimensional, with most women in the cohorts being compromised in one or more domains while few women scored highly in all three domains. Our analyses are limited by lack of data on adolescent exposures and on other relevant WHW dimensions such as safety, agency, empowerment, and violence. Further research is needed in LMICs for identifying and measuring the multiple domains of WHW.

Growing attention is being given by governments, international agencies, and donors to improving health and well-being [1,2]. Yet there is limited consensus as to what domains and constructs comprise good health and well-being [3] and on how these domains may relate to one another. For girls and women, it is essential to recognise how health and well-being are driven by gender inequity, including access to and control of resources, gender norms and stereotypes, and the gendered division of labour [4]. Gendered health behaviours can also influence health and well-being [5], as well as intersect with other systems of discrimination including racism, classism, ageism and homophobia to further deepen health inequities [5].

Although the original definition of health proposed by the World Health Organization (WHO) encompasses physical, mental, and social well-being [6], the literature on the health of girls and women in low and middle-income countries (LMICs) is heavily focused on reproductive and maternal outcomes as well as on specific diseases such as HIV and AIDS. These concerns are undoubtedly important but may divert attention away from more holistic constructs of health and well-being [7].

The operationalisation of women’s health and well-being (WHW) as a linked concept has faced enormous challenges. In 2014, the United Nations Development Programme (UNDP) reviewed composite measures of well-being that focus on ‘attainment in multiple and diverse aspects that impact on physical and mental quality of life’ [8]. Most of these measures encompassed political, economic, environmental, health, and social domains, with a focus on high-income settings [7,9,10]. However, these indices were not developed specifically for women, and generally fail to capture important but hard-to-measure concepts such as empowerment, agency, happiness, and mental health. Another omission is overlooking how health and well-being intersect with inequalities due to socioeconomic position and ethnicity [11,12]. Lastly, most existing approaches do not span the life course nor cover critical time periods, in addition to failing to consider how events in childhood and adolescence may shape adult health and well-being [13–16]. In particular, information on the pre-conceptional period and on older persons is seldom incorporated.

We studied WHW using data from the COHORTS consortium, which includes six long-running birth cohorts in LMICs with at least 1000 members at recruitment and with frequent visits in early life and follow up into adulthood [17]. These population-based cohorts were launched from 1969 to 1993, and in the most recent follow-up visits women were aged 22 years or older. We had two main objectives. The first was to investigate how relevant variables are clustered in adult women, and to explore different domains of WHW. The second objective was to examine how these domains are related to social, environmental, nutritional, and developmental conditions women may have faced in their early years.

Our analyses are part of the project entitled ‘Women’s health and well-being exemplars consortium’. A life-course approach is being used to capture health and well-being indicators across the age continuum [13–16]. Systematic reviews and analyses of existing data from LMICs led to a conceptual framework with 10 dimensions of WHW derived from Sen’s capabilities approach [18]. The dimensions include education, environment, health, empowerment and agency, safety and security, harmful practices and gender-based violence, poverty, nutrition, access to services, and work. This framework is being used to select exemplar countries for in-depth studies aimed at identifying the main drivers of progress for women [19].

## METHODS

### Participants and measurements

The COHORTS consortium includes six large population-based birth cohorts from LMICs with at least 22 years of follow up [17], from five countries: Brazil (two cohorts), Guatemala, India, Philippines and South Africa. Due to lack of information on child development and adult intelligence, it was not possible to include the Indian cohort in the present analyses. **Table 1** provides a brief description of each cohort. Since there was substantial variability in the age of participants in the INCAP study, we provided the entire age range for this cohort. The wide range is because recruitment included children under seven years of age at the start of the trial as well as those born during the trial period [20]. Detailed information on age at outcome measurement is described in Table S1 in the **Online Supplementary Document**.

**Table 1:** Characteristics of the six birth cohorts

Study	Cohort inception	Who was enrolled?	Age (years) at the latest adult examination and number* of women
Pelotas Birth Cohort, Brazil	1982	All hospital births in the city. All social classes.	30 (1737)
Pelotas Birth Cohort, Brazil	1993	All hospital births in the city. All social classes.	22 (1661)
INCAP Nutrition Trial Cohort, Guatemala	1969–77	Nutrition trial in 4 villages. Rural, poor.	40–57 (596)
Cebu Longitudinal Health & Nutrition Survey, Philippines	1983–84	33 randomly selected communities of Metro Cebu; 75% urban. All social classes.	34 (481)
Birth to 30, South Africa	1990	Soweto, Johannesburg. Predominantly urban poor, ‘Black’.	28 (475)

*Sample sizes reflect the number of participants with information on all outcome variables used in principal components analysis.

Four groups of variables were studied: family resources at birth, child growth and development, human capital stocks at around age 20 years, and adult WHW measures (detailed below). Therefore, the data used in this study covers a wide period, from birth (for perinatal variables) until adulthood (for outcome variables). The start date of the recruitment period is therefore the first birth in each cohort: 1 January 1969 for the INCAP cohort; 1 January 1982 for the 1982 Pelotas cohort; 1 May 1983 for the Cebu cohort; 23 April 1990 for the Soweto cohort; and 1 January 1993 for the 1992 Pelotas cohort. The end date of the recruitment period is when the last follow-up visit ended: 28 February 2013 for the Pelotas 1982 cohort; 31 July 2016 for the Pelotas 1993 cohort; and 30 June 2018 for the other three cohorts. The data analysed in the present analysis were accessed in 1 July 2021.

#### Family resources at birth

Maternal schooling. Information collected when the cohort member was born; expressed in years completed with a pass grade.

Maternal height. Measured by the study teams in the postnatal period; expressed in cm.

Socioeconomic position in early life was assessed using household income in the Cebu and Pelotas (1982 and 1993) cohorts. In Guatemala and Soweto, wealth indices were calculated based on household assets and building characteristics of their residence and expressed in quintiles [21].

Water and sanitation. Collected up to two years of age of the participants. Two variables were measured: toilet facilities, classified as flush or other; and water supply, classified as either improved (from pipe or pump; unshared in Pelotas, Soweto and Cebu; shared in Guatemala) or unimproved.

Birth order. Collected soon after birth by interviewing the mother, based on her previous number of liveborn infants (including the cohort member).

#### Child growth and development

Birthweight. Measured by the study team soon after birth; continuous variable expressed in multiples of 100 g.

Length-for-age and weight-for-age (Z scores) at two years. Children were weighed by the research teams at around two years of age (one year of age in the 1993 Pelotas cohort), and their height (in South Africa) or recumbent length (other sites) were measured. Z scores were calculated using the WHO Growth Standards.

Development quotient (Z scores) at 4–8.5 years. Children were tested by the research teams between the ages of four and eight and a half years, and their scores were standardised within each site to Z scores. The Cebu cohort (average age of eight and a half years) used the Philippine Nonverbal Intelligence Test (PNIT), modelled on the Raven’s Colored Progressive Matrices. The Guatemala cohort (4–7 years of age) used a Preschool Battery consisting of 22 sub-test tests, drawn from a variety of sources including the Wechsler Preschool and Primary Scale of Intelligence (WPPSI). When data were not available for a child at the age of seven years, results obtained at the closest age (six or five years) were used. The 1982 and 1993 Pelotas cohort (four years of age in both) used the Griffiths Scales and the WPPSI, respectively. The Soweto cohort (five years) used 32 items from the Revised Denver Pre-screening Questionnaire (R-DPDQ) covering personal-social, fine motor, gross motor, and language abilities.

#### Human capital stocks

Attained schooling (years), expressed in number of years of schooling completed with a pass grade.

IQ (harmonised units). The research teams administered the Wechsler Adult Intelligence Scale (Pelotas 1982 and 1993 cohorts) and Ravens Progressive Matrices (Cebu, Guatemala and Soweto), both standardised within each site, with a mean of 100 and standard deviation of 15 points.

Height (cm), measured by the study team and expressed in cm.

Teenage childbearing (yes/no): having had a child before their 20th birthday.

#### Adult women’s health and well-being

Psychological symptoms score (points) – number of symptoms reported in the Self-Reported Questionnaire (SRQ), ranging from zero to 20 symptoms [22].

Happiness Scale. Answer to the following question: ‘On a scale from 1 to 5, with 1 being very unhappy and 5 being very happy, how would you rate yourself, in general?’ In Pelotas, the scale ranged from 1 to 7, so this variable (represented by V) was scaled as follows: 2/3 × (V – 1) + 1, so as to range from 1 to 5.

Overweight/obesity. Adult weight and height were measured by each study team and body mass index (BMI) was calculated in kg/m^2^; overweight or obesity was defined as values of 25 or higher. Overweight and obesity were treated as a single outcome for the sake of simplicity.

Underweight. Women with BMI below 18.5 kg/m^2^ were classified as being underweight.

Metabolic syndrome score (0–5 points). Number of signs presented by each woman, including: abdominal adiposity (waist circumference ≥88 cm); raised blood pressure (systolic blood pressure ≥130 mm of mercury (mmHg) or diastolic blood pressure ≥85 mm Hg or taking hypotensive medication); raised triglycerides (≥150 mg/dL or taking triglyceride-lowering medication); raised plasma glucose (≥100 mg/dL for fasting glucose (all except the Pelotas cohorts) or ≥200 mg/dL for random glucose or taking diabetes medication) and reduced high-density lipoprotein (HDL) (<50 mg/dL).

### Statistical methods

All analyses were restricted to women and were carried out for each cohort separately.

For data description, human capital stocks and adult health and well-being variables were dichotomised using cohort-specific median values for intelligence quotient (IQ) and height, full secondary school for schooling (except in Guatemala where primary school was used) 25 kg/m^2^ for BMI [23], two signs for metabolic syndrome [24], seven symptoms on the SRQ [25], and an arbitrary score of five out of seven (or four out of five) on the Happiness Scale.

We performed a series of analyses to derive quantitative measures of WHW based upon an a priori conceptualisation of postulated domain of outcomes, based upon the available literature. The first domain was human capital, composed of IQ, schooling, height (which are well-established markers of human capital) and teenage childbearing (for which there is strong evidence and theory linking to lower human capital – for example, by affecting schooling). The second domain was metabolic health, composed of BMI (an established predictor of cardiometabolic health) and metabolic syndrome score (a composite of signs of cardiometabolic illness). The third domain was psychological health, composed of happiness and SRQ, which are strongly and likely bidirectionally linked. Initially, we generated Pearson correlation matrices to confirm whether the variables theoretically belonging to the same dimension of WHW were statistically related. We then used our conceptual considerations and the correlation results to inform formal principal component analysis (PCA), a data reduction technique aimed producing a small number of dependent variables for further analyses. More specifically, our main analysis simply applied PCA to each group of variables separately, and we used the first principal component as a summary quantitative measure which we refer to as WHW component of the corresponding WHW domain. Doing so only reduces the number of outcomes to be considered (three instead of eight), but does not force the summary measures of each WHW to be independent. We also performed a series of sensitivity analysis. We used continuous outcome variables for the correlation and PCA analyses, with the exception of teenage childbearing (a dichotomous variable) for which we used point-biserial correlations with the other outcomes.

We then performed association analyses to identify early-life predictors of the above-derived WHW components. In supplementary analyses, we also analysed the association between each of the eight WHW outcomes and early-life factors. The analyses were performed using regression models: linear regression for continuous or quasi-continuous outcomes (height, IQ, schooling in years, psychological symptoms, and well-being components) and Quasi-Poisson regression for binary (teenage childbearing and overweight/obesity) and count (metabolic syndrome signs and happiness score) outcomes. For Guatemala, all analyses included adjustment for year at birth given the wide range of this variable. Because the Guatemala study was originally a cluster-randomised trial, we also included a binary indicator of intervention or comparison group as a covariate. Given the importance of ethnicity as a distal determinant in the Pelotas cohorts, skin colour (self-reported as black, brown or white) was included as a covariate in all adjusted models for these two cohorts. Since the 1993 Pelotas Birth Cohort collected data in subsamples enriched for low birthweight cases at ages one and four years, all analyses in this cohort involving one or more variables collected at one or more of these ages (more specifically, length-for-age and BMI-for-age, and cognitive scores in childhood) were weighted using inverse sampling weights. All outcomes were coded so that higher values represent better health and well-being.

Covariates for statistical adjustment were selected based on the conceptual model that family resources at birth are the more distal determinants of WHWB, since they may influence child growth and development, human capital stocks, cardiometabolic and psychological health. Child growth and development measures were considered the more proximal determinants of WHWB, since they are posterior to family resources at birth. Under this model, each exposure-outcome association was adjusted for all more distal potential determinants of WHWB, as well as for selected variables in the same group (i.e. some family resources indicators were adjusted for selected family resource indicators; the same for child growth and development measures, which were also adjusted for all family resource indicators). A full description of which adjustment variables were included in each model is available in Table S2 in the **Online Supplementary Document**.

Adjustment was performed using multivariable regression and inverse probability of treatment weighting simultaneously. Additional sensitivity analyses were performed to assess the impact of missing covariate data, but this had no material effect on the results.

Cohort-specific results were combined using weighted random effects meta-analysis, where each cohort received the same weight. The exception was the two Pelotas cohorts, which had weights that add to one. This ensures that the pooled point estimate is a simple average of all sites (with the two Pelotas cohorts counting as one site), with the width of the confidence intervals incorporating between-cohort heterogeneity (quantified using the *I^2^* statistic and Cochran’s Q test). All analyses were performed using R 4.1.2 (https://www.r-project.org/).

## RESULTS

### Descriptive analyses

Up to 7300 women were included in the analyses of adult outcomes. The two Pelotas cohorts had close to 2000 women each, compared to around 700 in each of the other three cohorts (Table S3 in the **Online Supplementary Document**).

Histograms showing the distributions of the eight WHW outcomes in each cohort are presented in Figure S1 in the **Online Supplementary Document**, while the proportions of women who were above the cutoff points for each trait are shown in **Table 2**. Secondary education was completed by half to three quarters of the women, except in Guatemala where only 11% of the women had 12 or more years of schooling. For this reason, in this cohort this variable refers to primary schooling, which was completed by 43% of the women. Women from Guatemala and Cebu had lower mean height than in the other cohorts (Figure S1 in the **Online Supplementary Document**). Teen childbearing was highest in Guatemala, where almost half of all women delivered a child before the age of 20 years, and least common in Cebu and Pelotas 1993. Results for BMI and metabolic signs must be interpreted considering the average ages at which these outcomes were measured given that they tend to increase with age. Both overweight and metabolic signs were most frequent in Guatemala – the oldest cohort – where prevalence was close to 80% for both indicators, and least common in the youngest cohorts of Soweto and Pelotas 1993. The prevalence of underweight (BMI below 18.5 kg/m^2^) ranged from 0.9 in Guatemala to 6.4% in Soweto. Psychological symptoms were least prevalent in Cebu, and most frequent in Soweto. Happiness scores were similar in all cohorts, where two thirds to three quarters of women reported being happy most of the time. **Table 2** shows the proportions of women in each cohort will all eight positive attributes, which ranged from 0.9 in Guatemala to 10.8% in Pelotas 1993.

**Table 2:** Percent of women with positive attributes by cohort

Attribute	Cebu	Guatemala	Pelotas 1982	Pelotas 1993	Soweto	*P*-value
IQ:>cohort-specific median	48.2	48.9	45.2	48.6	49.2	0.178†
Schooling:≥full secondary	68.1	42.9*	48.6	64.7	72.1	<0.001
Height:>cohort-specific median	49.7	50.0	49.6	49.4	49.7	0.999†
No teenage childbearing	79.8	56.4	73.9	85.2	66.4	<0.001
BMI: <25 kg/m^2^	52.6	21.3	46.4	56.8	53.7	<0.001
Metabolic signs: ≤2	79.0	20.4	95.2	94.3	93.7	<0.001
SRQ: ≤7 symptoms	87.4	78.0	72.9	75.9	55.2	<0.001
Happiness: ≥4 or ≥5	75.9	77.0	71.7	72.9	67.3	<0.001
All traits	8.1	0.9	8.0	10.7	3.8	<0.001
IQ:>cohort-specific median	48.2	48.9	45.2	48.6	49.2	0.178†

BMI – body mass index, IQ – intelligence quotient, SRQ – Self-Reported Questionnaire

*Full primary education in Guatemala.

†Since cohort-specific medians were used as cut-offs, the *P* values were expected to be high.

### Correlation and principal component analyses

We used the continuous scores for each indicator (except teen childbearing, a binary variable) to calculate pairwise Pearson correlations within each cohort as well as for all cohorts combined (Figure S2 in the **Online Supplementary Document**). In all sites, three consistent clusters of variables became evident: IQ and schooling (referred to as human capital); BMI and metabolic signs (metabolic health); and SRQ and happiness (psychological health). Results for height and teenage childbearing were not fully consistent across the cohorts. Height was positively correlated with IQ and schooling in four cohorts, the exception being Soweto where height was virtually uncorrelated with any other variable. Lack of teenage childbearing was also positively correlated with IQ and schooling in four cohorts, with Cebu being the exception. In Guatemala, women who did not become a mother as teens had lower BMI and fewer metabolic signs. In the pooled analysis, both height and lack of teenage childbearing were positively but weakly correlated with IQ and schooling.

We used these correlation results to develop three separate PCA models. First, we applied PCA to IQ, schooling, height and teenage childbearing and extracted the first component, which we interpret as a measure of human capital. A similar process was performed for BMI and metabolic signs to extract the metabolic health component, and for SRQ and happiness to extract a psychological health component.

**Figure 1:**
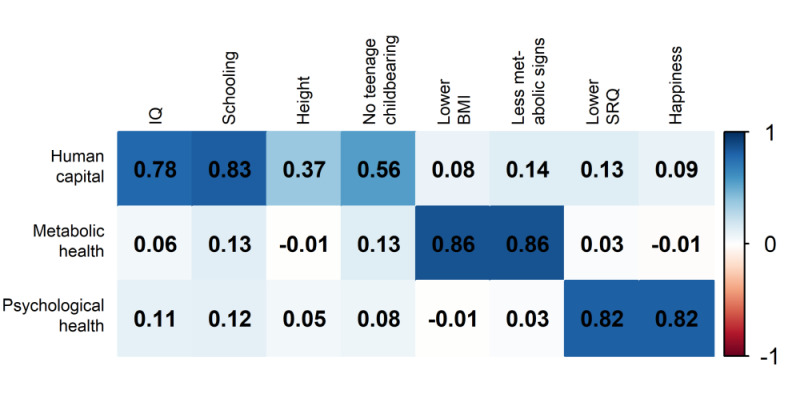
Pairwise Pearson correlation coefficients of the three separately generated PCA components with well-being outcomes in the pooled analyses from the five cohorts. PCA – principal component analysis

Initially, the three separate PCA models were performed within each cohort. For estimating the first component we carried out sensitivity analyses by including and excluding the height and teen childbearing variables (Figure S3 in the **Online Supplementary Document**). With the above-noted exceptions for height in Soweto and teen childbearing in Cebu, results were very robust and consistent. We therefore retained these two variables in the first component for the pooled analyses (i.e. PCA using the pooled data set containing all cohorts) (**Figure 1**). The amount of variance in the human capital variables explained by its first principal component was 44%. The corresponding figures for metabolic and psychological health were 75 and 67%, respectively. Pearson correlation coefficients were equal to 0.13 between human capital and metabolic health components, 0.14 between human capital and psychological health, and 0.01 between metabolic and psychological health. The results confirm the existence of three distinct and virtually independent components of well-being. The weak or absent correlations among the three components are illustrated in Figure S4 in the **Online Supplementary Document**, which shows that only 5.8% of the women are ranked on the top tercile of all components and 33.2% were not in the top tercile for any of the three components.

Sensitivity analyses included running five PCAs and generating cohort-specific components and the results were virtually identical to those below (Figure S6 in the **Online Supplementary Document**), with correlations coefficients between the cohort-specific and corresponding pooled PCA components being ≥0.97 in all cohorts. As part of further sensitivity analyses, we ran a single PCA model including all eight outcome variables, and the above results were virtually unchanged (Figure S5 in the **Online Supplementary Document**). Correlation coefficients between each component generated separately in the three PCAs and the component generated or in the model with the eight outcomes were all above 0.97, showing that both approaches produced very similar results.

### Associations between early-life conditions and WHW components

**Figure 2:**
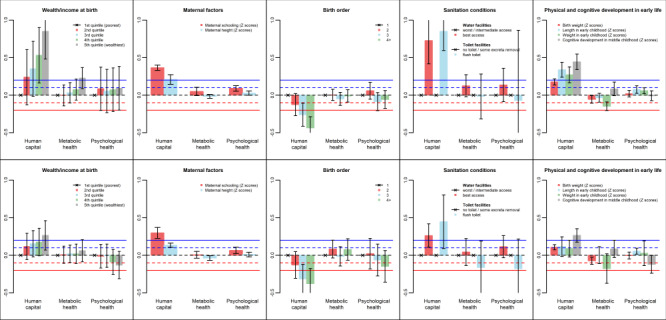
Linear regression coefficients (y-axis) of the three well-being components according to early life variables (unadjusted models on the top row and adjusted models on the bottom row). The coefficients are expressed as standard deviation changes in the well-being components associated with a standard deviation increase of continuous predictors or the mean difference (in standard deviation units) of the well-being components comparing every level of categorial predictors with its reference level (indicated by × ).

Having defined WHW components, we investigated their associations with early-life conditions using linear regression (**Figure 2**). For each exposure, we show crude and adjusted associations with the three WHW components. Detailed results, including point estimates, 95% confidence intervals and *P* levels for cohort-specific and pooled analyses for the 11 outcomes (eight separate variables and three domains) are provided in Table S5 in the **Online Supplementary Document**. We interpreted the results for the three domains in terms of effect sizes and confidence intervals in the crude and adjusted models (**Figure 2**). For brevity, we focus on the most robust results (those with *P* < 0.05). Table S4 in the **Online Supplementary Document** summarises the findings on effect sizes and confidence intervals.

Wealth or income at birth showed a strong, positive dose-response association with the human capital component in the crude analyses. This association persisted after adjustment for maternal age and parental education, despite being attenuated as effect sizes were reduced by almost half. Wealth or income at birth was also positively associated with the metabolic health component, but only in the crude analysis. There was no statistical evidence of associations with the psychological component.

Maternal schooling was positively associated with human capital and psychological health in the crude and adjusted analyses (with little evidence of attenuation). There was no association between maternal education and metabolic health. Maternal height was positively associated with human capital in both sets of analyses, weakly and inversely associated with metabolic health only in the adjusted analyses and not associated with psychological health. The presence of attenuation in the adjusted analyses for wealth, education and height may be explained by collinearity among these variables, as shown in Figure S2 in the **Online Supplementary Document**.

Birth order was inversely associated with human capital in the crude and adjusted analyses, but not with the other two well-being components. Poor water and sanitation were also associated with lower human capital in the crude and adjusted analyses even though the associations were greatly attenuated after adjustment, with reductions in effect sizes of about 50% (**Figure 2**). There was no strong statistical evidence of associations with the metabolic or psychological components.

Three indicators of growth in early life (birthweight, length and weight at the age of two years) were positively associated with the human capital component, although the association of weight at two years disappeared after adjustment, and the association with length was attenuated with effect size dropping from 0.34 (95% CI = 0.24, 0.44) to 0.12 (95% CI = −0.01, 0.25) units. Also, after adjustment, metabolic health was inversely associated with birthweight and weight at age two years (for the latter, confidence intervals in the adjusted analysis included the null, but this was due to lower precision rather than point-estimate attenuation), but not with length at age two years. Both length and weight in early childhood were positively associated with psychological health in the crude analysis, but only the association with length remained after adjustment. Cognitive development was positively associated with human capital in the crude and adjusted analyses and with psychological health in the adjusted analyses, but there was no association with metabolic health. It should be noted that data on birth length was not available in all cohorts and thus could not be analysed.

## DISCUSSION

Our analyses addressed two research questions: first, whether it would be possible to identify different dimensions or domains of WHW across different LMIC contexts; and second how early life factors may be associated with these domains. We addressed these questions by studying over 7000 women followed up from birth until adulthood in five LMIC cohorts. Consistent results were found across the five cohorts regarding the identification of three domains (human capital, metabolic and psychological health), on the outcome variables included in each domain and on their differential associations with early-life exposures. Women’s health and well being components that were generated separately for each domain were almost identical to those generated in a single model that included all outcomes. In addition, restriction of the analyses to those without missing data for any outcome variable also resulted in very similar results (data not shown). The robustness of our results is supported by the consistency of findings across the five cohorts, located in South and Central America, Africa and Oceania.

Using data on eight measures of WHW, our correlation and PCA analyses identified three independent components. The first, which we refer to as human capital or stocks includes IQ, schooling and height, which reflect the education and nutrition dimensions of the traditional definition of human capital [26] as well as absence of teen childbearing which is particularly important for the health and well-being of the women themselves [27] and of their offspring [28]. The second domain – metabolic health – included BMI and indicators of metabolic syndrome while the third factor – psychological health – included self-reported happiness and psychological symptoms. Virtually all associations among outcome variables were in the expected directions and robust results were obtained using different analytical strategies.

Our finding that the three domains are largely independent suggest that it may be difficult to arrive at a single comprehensive measure of WHW, as only about one in 17 women (5.8%) in the cohorts were classified in the upper terciles of all three components, while one third of them were not in the top tercile for any component. These proportions are close to what would be expected purely on mathematical terms given the lack of association among the three components. The independence between the human capital and metabolic domains is consistent with the observation that human capital indicators improved in many LMICs during the 2010s [29] in contrast with the increased risk for non-communicable diseases in most such countries [30]. Analyses of the mothers of the Cebu cohort members had also found that self-reported well-being was not closely related to physical health at ages 46 to 79 years, similarly to what we are now reporting for their daughters [31]. Independence between WHW domains is also supported by the diversity of our findings on their associations with early-life exposures. All nine exposures were associated with human capital in the expected direction, but associations with the other two domains were inconsistent. For metabolic health, there were inverse associations with birth weight and – to a lesser extent – with weight at the age of two years, whereas cognitive development in mid-childhood was associated with better metabolic health. For psychological health, only one association was observed with early-life exposures, namely a positive association with maternal education. The specificity of potential early-life determinants supports the notion that the three WHW domains are indeed independent.

Earlier analyses of data from the COHORTS had shown the negative impact of exposure to early-life poverty and child undernutrition on human capital indicators for adult men and women [27,32,33]. The association between early-life stature and adult IQ was shown to be mediated by childhood IQ [34]. Recent analyses [27] showed that inverse associations between early-life socioeconomic position and adult psychological problems were present in the two Pelotas cohorts, but not in the other sites. Differential patterns by sex were observed for overweight and metabolic signs, with early poverty being protective among men, while the associations among women were not clear cut. These earlier analyses did not include adjustment for covariates. Additional evidence on the impact of early-life poverty on adolescent growth and development is provided by the Young Lives study in Ethiopia, India, Peru, and Vietnam [35,36].

Previous analyses of COHORTS data had shown a strong positive association between maternal education and child development [37], but results on adult outcomes had not been published. Strong associations of maternal education with both human capital and psychological components support the intergenerational transmission of WHW [38].

Similarly to the present analyses, earlier COHORTS analyses found that birthweight was not associated with high blood pressure but was directly associated with BMI, which was largely explained by lean body mass [39–41]. In agreement with the present results, the earlier analyses showed that rapid weight gains in early life were inversely associated with adult metabolic health [39,42]. When comparing the present and earlier results, it should be noted that the former refers to the full sample with both sexes in five cohorts (including the Delhi cohort, but not the Pelotas 1993 cohort), and to outcomes collected at younger ages than reported here. It is also important to stress that the metabolic component includes blood pressure, BMI and metabolic signs, whereas earlier publications treated these conditions separately.

The main limitation of our analyses is the lack of information across all cohorts on additional important aspects of well-being such as agency, empowerment and violence. Such information is available from recent follow-up visits for some of the cohorts and deserves further analysis. It is also possible that the data we had did not cover all important aspects of the domains we studied. For example, soft skills are likely an important component of human capital and psychological health is a complex domain that might be hard to capture in detail. However, the fact that results were consistent across sites suggests that the available data were sufficient to capture at least a substantial portion of these domains. Second, socioeconomic gaps are wider in the two Pelotas cohorts than in the other studies, and this may have affected the results on early-life exposures leading to stronger associations with human capital indicators than those observed elsewhere [27]. There was some heterogeneity across cohorts in the magnitude of associations, but it is reassuring that similar components were observed in all cohorts, and that the strong associations of early determinants with human capital were also present in all cohorts. Another limitation is the lack of information on adolescent exposures, which precluded the analyses of how adolescent health, development and behaviours relate to the three components of adult well-being. This is particularly relevant for psychological and metabolic health, which in the present analyses showed weak associations with early-life exposures. As in all long-term cohorts involving examinations, particularly in LMICs, losses to follow up may be substantial, as discussed in detail elsewhere [17]. Another limitation is that the age of cohort members at the most recent follow up ranged from 22 to 57 years (**Table 1**), a difference that needs to be considered when interpreting the prevalence of metabolic and psychological outcomes (**Table 2**). However, these differences would not be expected to affect the correlations and PCA based on continuous outcome variables as for most of the present results. Lastly, our approach to characterise WHW domains does not address issues of causality. For example, low schooling is associated with teen childbearing, but the data do not allow assessment of temporality nor of the direction (if any) of a causal association. It should also be noted that, as part of a broad project on WHW, we restricted all analyses to cohort females. Further analyses may elucidate how the domains obtained for men differ from those being reported here.

## CONCLUSIONS

Summing up, our analyses indicate that WHW has at least three domains that are largely uncorrelated – with only one in twenty women performing well in all three components – and likely present different aetiologies. We believe this is the first set of analyses from LMICs on how different domains relate to one another, and on their differential association with early exposures. We hope that the present findings will contribute to identify what domains are essential for promoting the good health and well-being of women [3], and on how much these domains overlap with one another. In terms of interventions to ensure health and well-being, our findings point out three main avenues. First, they suggest that a variety of programmatic approaches are required, given the relative independence among domains. Second, they support recent pleas for early-life interventions, by putting children and adolescent girls at the centre of the global development agenda. Third, given the role of socioeconomic, educational and environmental factors, our results point to the importance of gender-sensitive, intersectoral interventions to reach those girls and women who are being left behind [43,44].

## Additional material


Online Supplementary Document
