# Supplementary material for: Women’s health and well-being in five birth cohorts from low- and middle-income countries: Domains and their associations with early-life conditions
Source: J Glob Health. 2024 Aug 16;14:04137. doi: 10.7189/jogh.14.04137 (PMC11327850; doi:10.7189/jogh.14.04137)
Supplement: Online Supplementary Document [file jogh-14-04137-s001.pdf]

Supplementary Material

Table of contents

Table S1. Mean age (years) at outcome measurement, by cohort. 2

Table S2. Covariates used in the adjusted analyses. 3

Table S3. Number of women with information on adult outcomes, by cohort. 4

Table S4. Summary of findings on effect sizes and 95% confidence intervals. 5

Figure S1. Histograms showing distributions of the eight indicators in the five cohorts. The vertical axis indicates the number of individuals in each range. 6

Figure S2. Pairwise Pearson correlation coefficients among outcome variables. 7

Figure S3. Pairwise Pearson correlation coefficients of wellbeing components from pooled analyses with outcomes, based on three separate PCA models. 8

Figure S4. Overlap of women classified in the upper tercile of each wellbeing component in the pooled analyses. 9

Figure S5. Pairwise Pearson correlation coefficients of wellbeing components from pooled analyses with outcomes, based on a single PCA model including the eight outcome variables. 10

Figure S6. Pairwise Pearson correlation coefficients between wellbeing components from pooled (p) and cohort-specific (c) analyses. 11

Table S1. Mean age (years) at outcome measurement, by cohort.

Trait	Cebu	Guatemala[A]	Pelotas 1982	Pelotas 1993	Soweto
IQ	34	40-57	30	18	28
Schooling	34	40-57	30	22	28
Height	21	38-55	30	22	22
Weight	34	38-55	30	22	22
Waist circumference	34	38-55	30	22	22
Blood pressure	34	38-55	30	22	22
Triglycerides	21	38-55	30	22	22
Plasma glucose	21	38-55	30	22	22
HDL cholesterol	21	38-55	30	22	22
SRQ	34	40-57	30	22	28
Happiness	34	40-57	30	18	28

[A]Given the high variability in age in this cohort, the range rather than the mean is shown.

Table S2. Covariates used in the adjusted analyses.

Exposure	Covariates[A,B]
Wealth/income	Maternal age, maternal schooling, paternal schooling
Maternal education	Maternal age, wealth/income
Maternal height	1: Maternal age, maternal schooling, paternal schooling, wealth/income
Birth order	2: 1 + maternal height
Water facilities	2 + toilet facilities
Toilet facilities	2 + water facilities
Birth weight	3: birth order + water facilities + toilet facilities
Length in early childhood	4: 3 + birth weight
Weight in early childhood	5: 4 + length in early childhood
Cognitive development in middle childhood	6: 5 + weight in early childhood

[A]In both Pelotas cohorts, skin color was also included as a covariate.

[B]In Guatemala, year of birth and intervention status were included as covariates in both minimally-adjusted (which we refer to as “crude” in the main text for simplicity) and fully-adjusted analyses.

Table S3. Number of women with information on adult outcomes, by cohort.

Trait	Cebu	Guatemala	Pelotas 1982	Pelotas 1993	Soweto
IQ	612	749	1872	2070	734
Schooling	847	774	1883	2027	730
Height	958	812	2231	1889	772
Teenage childbearing	931	778	1856	2091	888
BMI	593	689	1798	1875	764
Metabolic syndrome	482	675	1778	1814	633
SRQ	611	705	1884	2017	737
Happiness	611	700	1885	2091	733

Table S4. Summary of findings on effect sizes and 95% confidence intervals.

Exposure	Model	Human capital	Metabolic health	Psychological health
Wealth/income quintiles at birth	Crude	Strong	Weak to Strong	Moderate to Strong
	Adjusted	Weak to Strong	Weak to Strong	Strong to Weak
Wealth/income quintiles at birth (trend)	Crude	Weak to Strong	Weak to Moderate	Weak to Moderate
	Adjusted	Weak to Moderate	Weak to Weak	Weak to Weak
Maternal schooling	Crude	Strong	Weak to Moderate	Weak to Moderate
	Adjusted	Strong	Weak to Weak	Weak to Moderate
Maternal height	Crude	Moderate to Strong	Weak to Weak	Weak to Weak
	Adjusted	Moderate	Weak	Weak to Weak
Water facilities at birth	Crude	Strong	Weak to Strong	Weak to Strong
	Adjusted	Moderate to Strong	Moderate to Strong	Weak to Strong
Toilet facilities at birth	Crude	Strong	Strong to Strong	Strong to Strong
	Adjusted	Weak to Strong	Strong to Moderate	Strong to Strong
Birth order	Crude	Strong	Weak to Weak	Moderate to Weak
	Adjusted	Moderate to Strong	Weak to Strong	Strong to Weak
Birth order (trend)	Crude	Weak to Strong	Weak to Weak	Weak to Weak
	Adjusted	Weak to Strong	Weak to Weak	Weak to Moderate
Birth weight	Crude	Moderate to Strong	Weak to Moderate	Weak to Weak
	Adjusted	Weak to Moderate	Weak to Moderate	Weak to Weak
Length in early childhood	Crude	Strong	Weak to Weak	Weak to Moderate
	Adjusted	Weak to Strong	Moderate to Moderate	Weak
Weight in early childhood	Crude	Moderate to Strong	Weak to Strong	Weak
	Adjusted	Weak to Strong	Strong to Weak	Moderate to Moderate
Cognitive development in middle childhood	Crude	Strong	Weak to Moderate	Weak to Weak
	Adjusted	Moderate to Strong	Weak to Strong	Weak to Strong

Notes: Blue indicates positive, and red indicates negative associations. The terms *weak*, *moderate* and *strong* indicate the confidence limits of the associations. For binary exposures (water and toiler facilities), *weak* implies that the confidence limit of the mean difference (in standard deviation units (SD) of the outcome) was between 0 and 0.1 (in absolute value); *moderate* if between 0.1 and 0.2; and *strong* if greater than 0.2. For categorical exposures (wealth/income quintiles and birth order), the same criteria were applied for the comparisons: 1st and 5th quintiles; and 1 and ≥4 birth order rank. For all other exposures, which are numeric, the same criteria were applied to effect estimates expressed as SD changes in outcome per SD increase in exposure.

For detailed results see the Excel appendix (Supplementary Table 5).

Figure S1. Histograms showing distributions of the eight indicators in the five cohorts. The vertical axis indicates the number of individuals in each range.

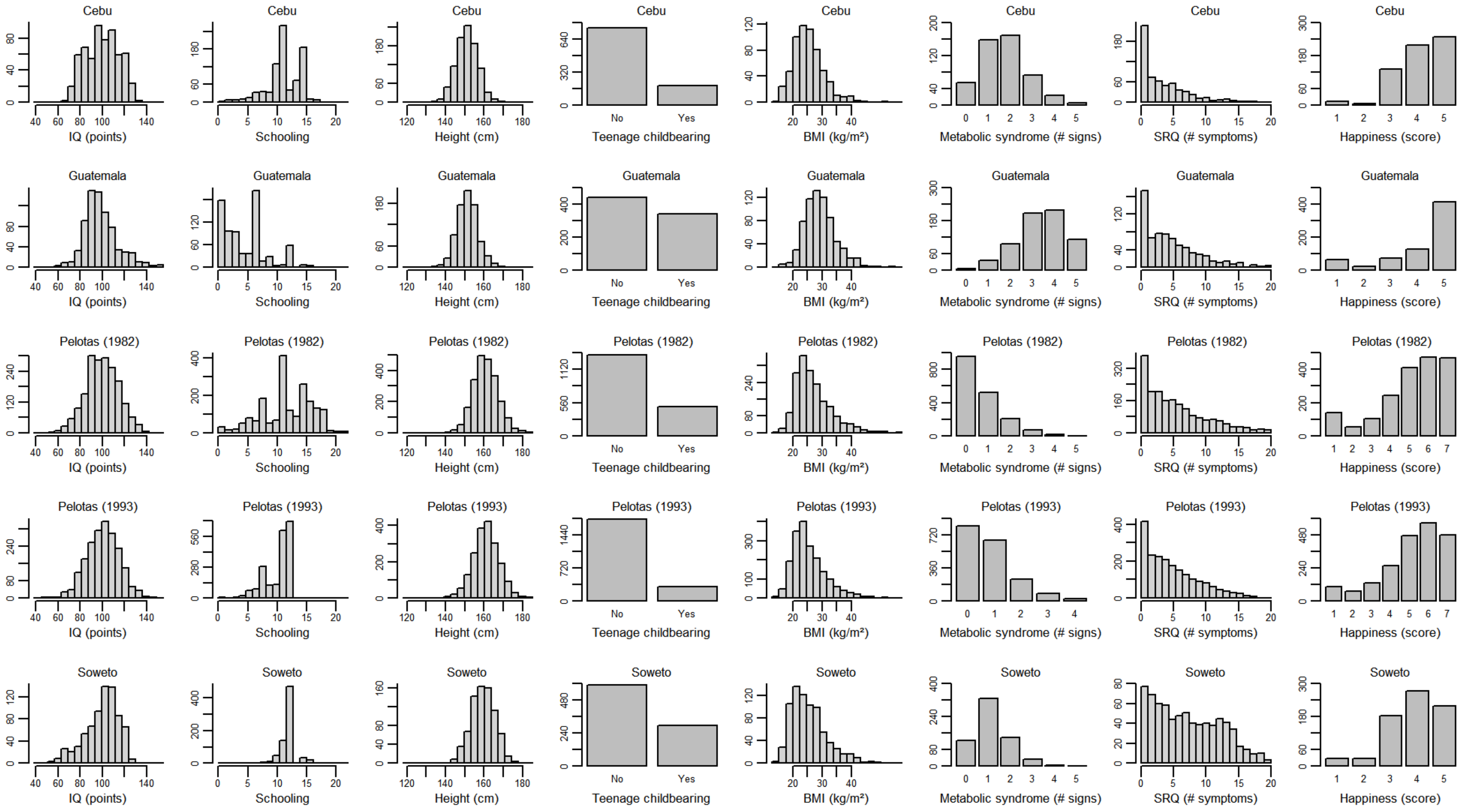

Figure S2. Pairwise Pearson correlation coefficients among outcome variables.

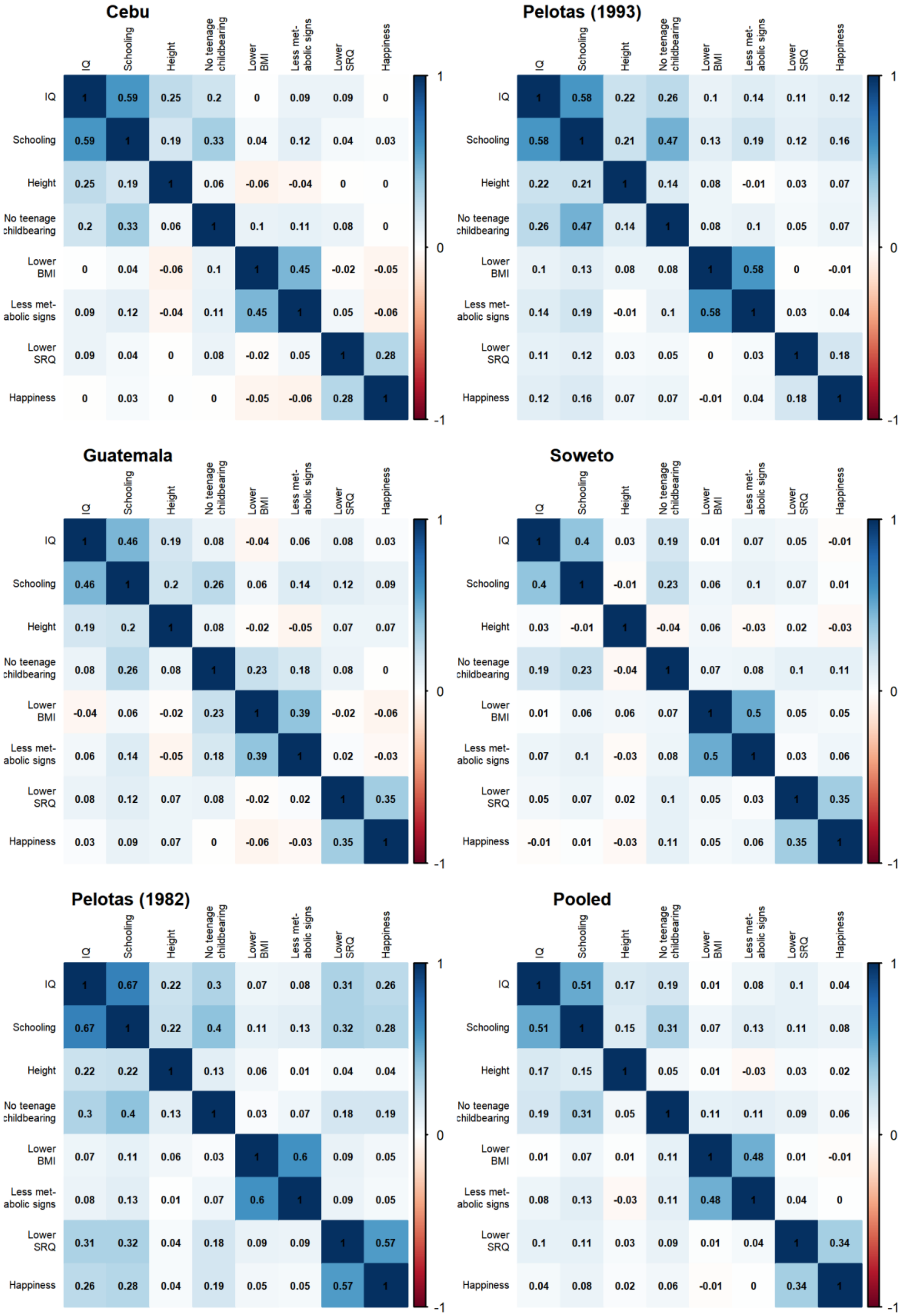

Figure S3. Pairwise Pearson correlation coefficients of wellbeing components from pooled analyses with outcomes, based on three separate PCA models.

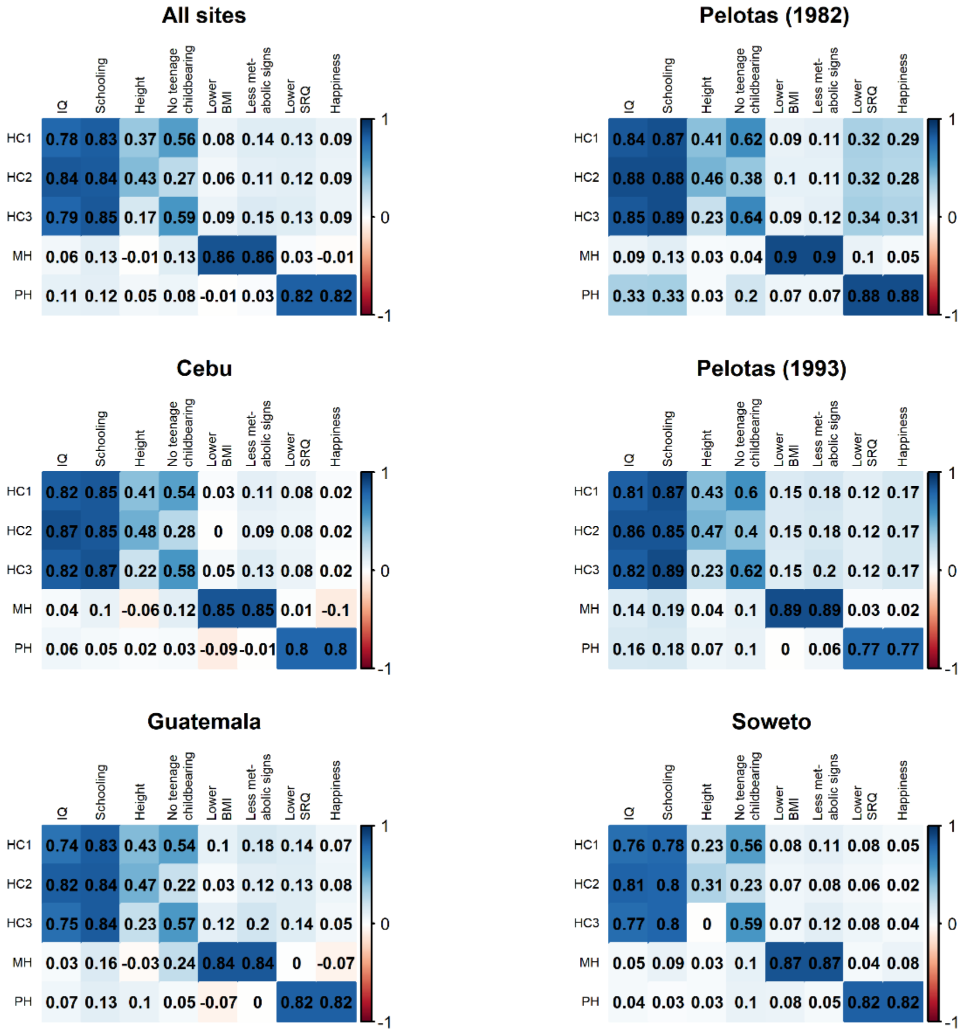

HC: Human capital (HC1: including both height and teenage childbearing; HC2: including only height; HC3: including only teenage childbearing).

MH: metabolic health.

PH: psychological health.

Figure S4. Overlap of women classified in the upper tercile of each wellbeing component in the pooled analyses.

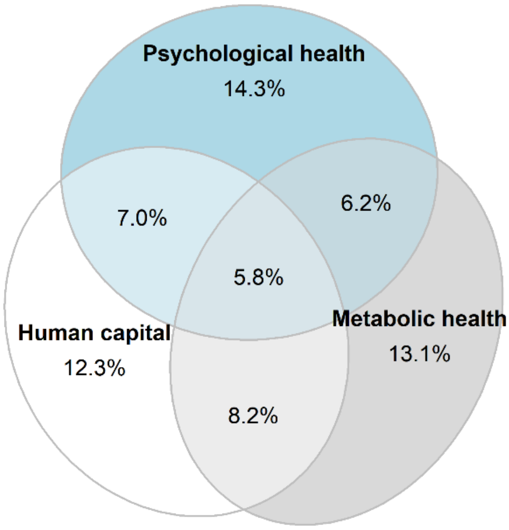

Figure S5. Pairwise Pearson correlation coefficients of wellbeing components from pooled analyses with outcomes, based on a single PCA model including the eight outcome variables.

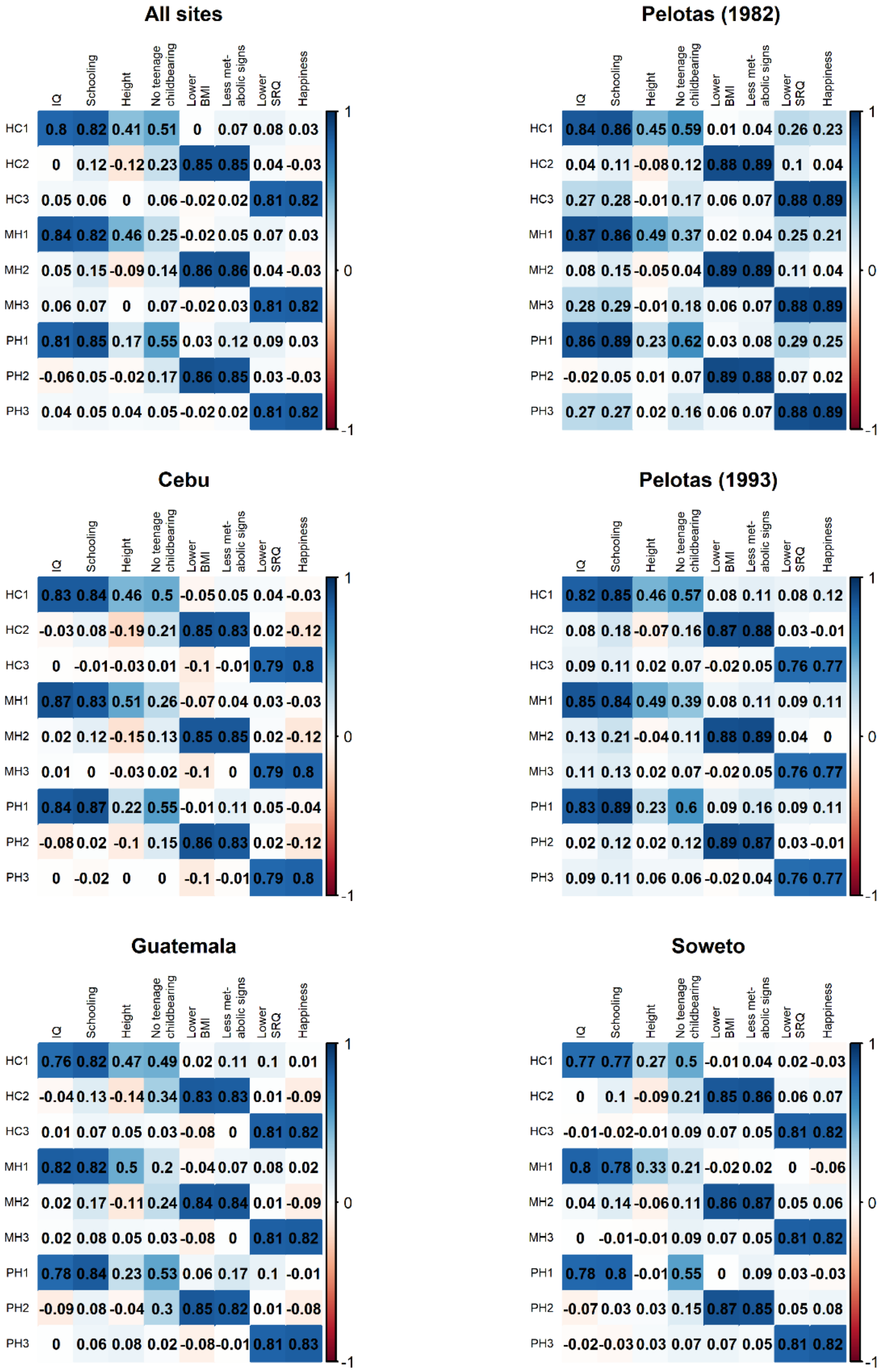

HC: Human capital. MH: metabolic health. PH: psychological health. 1: including both height and teenage childbearing; 2: including only height; 3: including only teenage childbearing.

Figure S6. Pairwise Pearson correlation coefficients between wellbeing components from pooled (p) and cohort-specific (c) analyses.

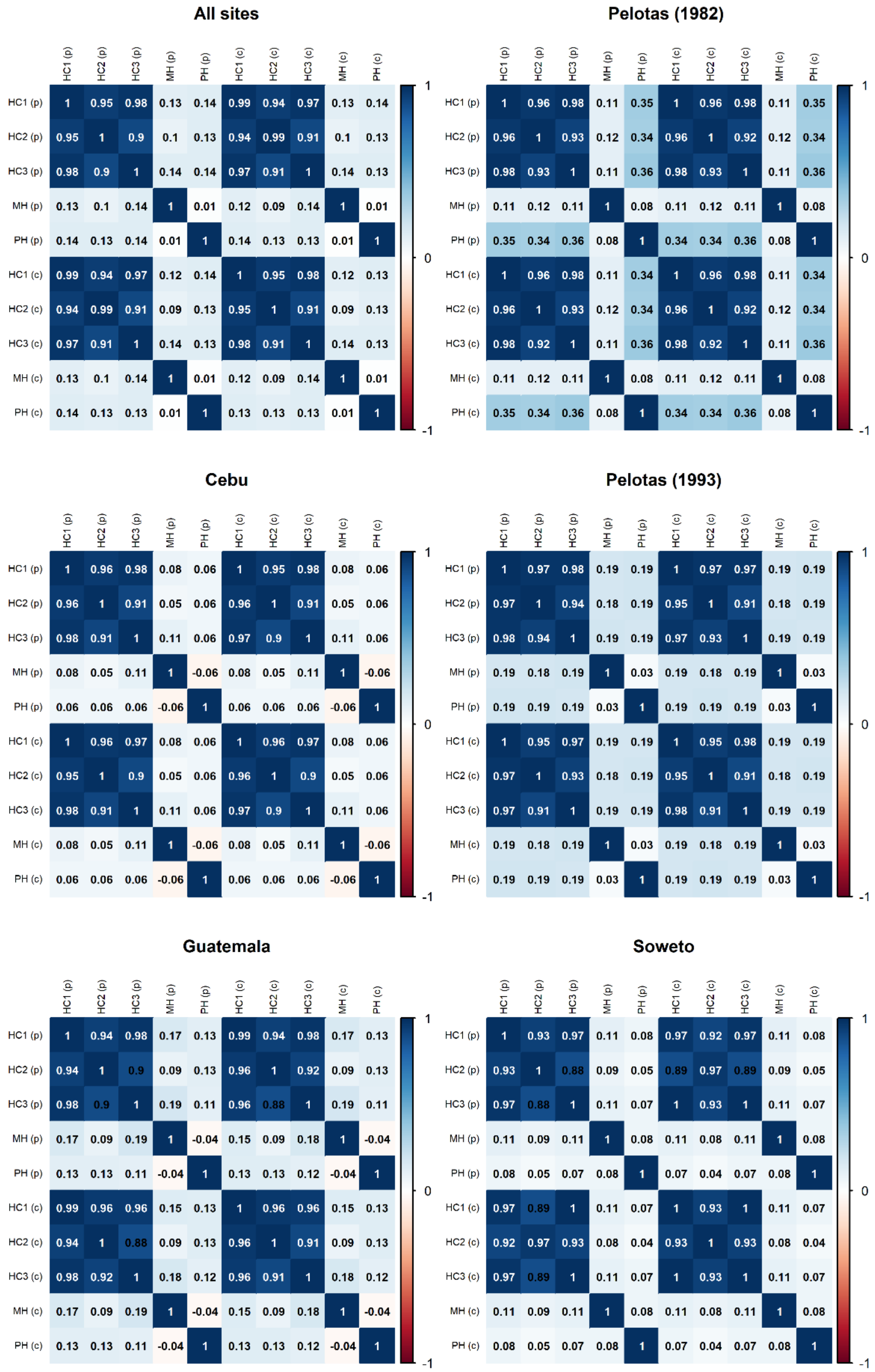

HC: Human capital (HC1: including both height and teenage childbearing; HC2: including only height; HC3: including only teenage childbearing). MH: metabolic health. PH: psychological health.
